# No evidence for age-related alterations in the marmoset retina

**DOI:** 10.3389/fnana.2022.945295

**Published:** 2022-09-02

**Authors:** Silke Haverkamp, Katja Reinhard, Leo Peichl, Matthias Mietsch

**Affiliations:** ^1^Department of Computational Neuroethology, Max Planck Institute for Neurobiology of Behavior—Caesar, Bonn, Germany; ^2^Retinal Circuits and Optogenetics, Centre for Integrative Neuroscience, University of Tübingen, Tübingen, Germany; ^3^Institute of Clinical Neuroanatomy, Dr. Senckenbergische Anatomie, Goethe University Frankfurt, Frankfurt am Main, Germany; ^4^Laboratory Animal Science Unit, German Primate Center, Göttingen, Germany; ^5^DZHK (German Center for Cardiovascular Research), Partner Site Göttingen, Göttingen, Germany

**Keywords:** primate retina, aging, ganglion cell loss, sprouting, ectopic synapses, common marmoset, photoreceptor degeneration, cellular reorganization

## Abstract

The physiological aging process of the retina is accompanied by various and sometimes extensive changes: Macular degeneration, retinopathies and glaucoma are the most common findings in the elderly and can potentially lead to irreversible visual disablements up to blindness. To study the aging process and to identify possible therapeutic targets to counteract these diseases, the use of appropriate animal models is mandatory. Besides the most commonly used rodent species, a non-human primate, the common marmoset (*Callithrix jacchus*) emerged as a promising animal model of human aging over the last years. However, the visual aging process in this species is only partially characterized, especially with regard to retinal aberrations. Therefore, we assessed here for the first time potential changes in retinal morphology of the common marmoset of different age groups. By cell type specific immunolabeling, we analyzed different cell types and distributions, potential photoreceptor and ganglion cell loss, and structural reorganization. We detected no signs of age-related differences in staining patterns or densities of various cell populations. For example, there were no signs of photoreceptor degeneration, and there was only minimal sprouting of rod bipolar cells in aged retinas. Altogether, we describe here the maintenance of a stable neuronal architecture, distribution and number of different cell populations with only mild aberrations during the aging process in the common marmoset retina. These findings are in stark contrast to previously reported findings in rodent species and humans and deserve further investigations to identify the underlying mechanisms and possible therapeutic targets.

## Introduction

The common marmoset (*Callithrix jacchus*) is a New World primate that in recent years has become increasingly popular for various research areas like behavioral science, neurobiology and visual neuroscience (Solomon and Rosa, 2014; Mitchell and Leopold, 2015). The introduction of genetically engineered models has broadened the use of this species in various research fields even further (Sasaki et al., 2009; Park and Silva, 2019). With an average lifespan of 9–12 years and a maximum of 22 years (Nishijima et al., 2012), the common marmoset represents the most short-lived species among the commonly used non-human primates. With a compressed lifespan in comparison to the Old World macaques, marmosets are fully mature by about 2 years of age and considered aged by 8 years (Abbott et al., 2003). The short life span among primates, together with several similarities to humans in various aging fields (neurodegenerative decline, immunological senescence, cardiovascular alterations, cartilage changes, occurrence of diabetes, etc.), an ease of handling and its small body size make the marmoset an attractive animal model for aging research (Tardif et al., 2011; Mietsch et al., 2020; Moussavi et al., 2020). In the present study, we have investigated whether this also holds for retinal aging in view of the following background.

In the human retina, neuronal cell loss is one of the hallmarks of aging. Photoreceptors decrease in density with increasing age and it seems that rods are more affected than cones (Gao and Hollyfield, 1992; Curcio et al., 1993; Panda-Jonas et al., 1995; Curcio, 2001). Cell loss also occurs in the ganglion cell layer (GCL). Some studies in humans showed an age-related decrease of the optic nerve fiber population (Balaszi et al., 1984; Johnson et al., 1987; Jonas et al., 1992), others found that the number of retinal ganglion cells in the GCL decreases with age (Gao and Hollyfield, 1992; Curcio and Drucker, 1993; Harman et al., 2000). Another aging phenotype is the occurrence of dendritic reorganization: Rod bipolar cells and ON cone bipolar cells, as well as horizontal cells, of the human retina were shown to undergo extensive dendritic reorganization during normal aging (Eliasieh et al., 2007). Extended growth of bipolar cell dendrites into the outer nuclear layer (ONL) occurred most abundantly in the peripheral retina, which is in contrast to the higher loss of rod photoreceptors in the central retina.

Marmoset retinas have been extensively studied. They exhibit cone photoreceptor densities resembling those of humans (Troilo et al., 1993). There also are detailed characterizations of retinal ganglion cell morphology (Ghosh et al., 1996; Moritoh et al., 2013; Masri et al., 2019; Nasir-Ahmad et al., 2022) as well as investigations of bipolar and amacrine cell populations (Weltzien et al., 2015) and calbindin expression in the inner retina (Chandra et al., 2019). However, with regard to aging-associated changes in retinal structures and the suitability of the common marmoset for such investigations only little is known: A recent study revealed the regular occurrence of age-related glaucoma-like degenerations associated with alterations of the Lamina cribrosa and ganglion cell complex (Noro et al., 2019). Pigment granules in the retinal pigment epithelium as well as alpha-synuclein and beta-synuclein were shown to change during the aging process (Hadrian et al., 2019). Proteomic analyses revealed signs of inflammation and a decrease in angiogenetic profiles in old marmoset retinas (König et al., 2019). Although these studies provided valuable insights into the structures, physiology and pathology of the aging marmoset eye, further characterizations especially with regard to the morphology and pathological changes of the retina during the aging process are necessary.

In the present study, we therefore aimed to characterize potential aging-associated retinal changes in the common marmoset. For this purpose, we performed extensive investigations of cell distributions (cell type labeling, foveal ganglion cell pattern, distribution of microglia), of cell death patterns (including ganglion cell loss), and of structural reorganization. We immunolabeled several retinal cell types in young and aged marmoset retinas and detected no age-related difference in the staining pattern. We quantified the number of photoreceptors and retinal ganglion cells and found no significant decrease or increase in the aged retina. Furthermore, cell death marker comparisons and foveal ganglion cell patterns revealed a stable situation. Finally, we also examined the sprouting behavior of rod bipolar cells in aged retinas and found only very few examples of outgrowing dendrites.

## Materials and methods

### Tissue collection and preparation

Marmoset retinal tissue used in this study was obtained from 10 adult/control (2–7 years) and 12 old/aged (8–15 years) clinically healthy common marmosets. The animals were sacrificed within a broad aging study at the German Primate Center in Göttingen, Germany. All procedures were approved by the institutional animal welfare committee and by the Lower Saxony State Office for Consumer Protection and Food Safety (reference number 33.19−42502−04−17/2496). Housing and husbandry conditions (for details see Mietsch et al., 2020) were in accordance with the law for animal experiments issued by the German government (Tierschutzgesetz) and complied with the European Union guidelines on the welfare of non-human primates used in research (EU directive 2010/63/EU). The animals were anesthetized intramuscularly with a combination of ketamine (50 mg/kg, Ketamin 10%, WDT), xylazine (10 mg/kg, Xylariem 2%, Ecuphar) and atropine (1 mg/kg, Atropinsulfat, Dr. Franz Koehler Chemie GmbH), and killed by an intraperitoneally administered overdose of pentobarbital (150–200 mg/kg). The eyes were enucleated, the right eye was immersion fixed in 4% paraformaldehyde (PFA) for 60 min at room temperature, and the left eye was used for physiological experiments unrelated to this study. Following fixation, the eyes were stored at 4°C in PBS and 0.02% sodium azide. For immunohistochemistry, the retinas were dissected from the eyecup and retinal pieces of defined eccentricities were used as a flatmount or sectioned vertically (60–100 μm) with a vibratome (Leica VT 1200 S).

Additionally, retinal human tissue was obtained from four donors, 42, 53, 79, and 89 years old (for detailed information about the human retinal donations see Reinhard and Münch, 2021). Retinal pieces were fixed with 4% PFA, cryoprotected in Tissue Freezing Medium (Leica Biosystems, Wetzlar, Germany), and frozen vertical sections of 12–20 μm were taken with a cryostat (Leica CM3050S) and collected on Superfrost Plus slides.

### Immunohistochemistry

Immunohistochemical analyses were performed on flat-mounted and sectioned tissue with the primary antibodies listed in Table 1. Antibodies were diluted in PBS, pH 7.4, containing 0.5% Triton X-100 and 0.02% sodium azide with 3% normal donkey serum. Immunolabeling was performed using the indirect fluorescence method. Cryostat and vibratome sections were incubated overnight in the primary antibodies, followed by a 1 h incubation in the secondary antibodies, which were conjugated to Alexa Fluor 488, Alexa Fluor 647 (Invitrogen), Cy3 or Cy5 (Dianova). In double-labeling experiments, sections were incubated in a mixture of primary antibodies, followed by a mixture of secondary antibodies. Whole mounts were incubated for 2–4 days in the primary and for 2 h in the secondary antibody solutions, containing 1% Triton X-100. The number of animals used for the different experiments and immunostainings is given in Table 2.

**TABLE 1:** Primary antibodies used in this study.

Antibody	Antigen	Host	Dilution	Source, cat#, RRID
Calbindin	CaBP-D-28 kDa purified from chicken gut	Mouse	1:1,000	Swant, 300, RRID:AB_10000347
Calbindin	Recombinant rat calbindin D -28k	Rabbit	1:2,000	Swant, CB-38, RRID:AB_10000340
CD15	U-937 Histiocytic cell line, purified from tissue culture supernatant or ascites by affinity chromatography	Mouse	1:100	BD Pharmingen, 559045, RRID:AB_397181
ChAT	Purified human placental choline acetyltransferase enzyme	Goat	1:200	Millipore, AB144P, RRID:AB_2079751
CtBP2	Mouse C-terminal binding protein 2, aa 361– 445	Mouse	1:5,000	BD Transduction, 612044, RRID:AB_399431
GlyT1	Aa 614–633 from cloned rat GlyT1	Goat	1:5,000	Millipore, AB1770, RRID:AB_90893
GNB3	Peptide with sequence SGHDNRVSCLGVT, corresponding to aa 309–321 of human transducin β chain 3	Goat	1:200	Aviva Systems Biology, OALA06860, RRID:AB_2909439
Iba1	17-kDa EF hand protein specifically expressed in macrophages/microglia	Rabbit	1:1,000	Wako, 019-19741, RRID:AB_839504
L/M-Opsin	Recombinant human red/green opsin	Rabbit	1:1,000	Millipore, AB5405, RRID:AB_177456
Parvalbumin	Recombinant rat parvalbumin	Rabbit	1:2,000	Swant, PV-28, RRID:AB_2315235
Parvalbumin	Clone PARV-19, ascites fluid	Mouse	1:10,000	Sigma, P3088, RRID:AB_477329
PKCα	Protein kinase C, regulatory subunit α; peptide sequence: KVNPQFVHPILQSAV	Rabbit	1:5,000	Sigma, P4334, RRID:AB_477345
RBPMS	KLH-conjugated peptide corresponding to a sequence from the N-terminal region of human RNA binding protein with multiple splicing (RBPMS)	Guinea pig	1:500	Millipore, ABN1376, RRID:AB_2687403
Rhodopsin 1D4	C-terminal nine amino acids of bovine rhodopsin known as the 1D4 epitope.	Mouse	1:200	Abcam, Ab5417, RRID:AB_304874
S-Opsin	Peptide corresponding to amino acids 1–50 from the N terminus of the opsin protein encoded by OPN1SW of human origin	Goat	1:500	Santa Cruz, sc-14363, RRID:AB_2158332
TH	Recombinant protein corresponding to aa 65–255 from human TyrH	Guinea pig	1:1,000	SySy, 213004, RRID:AB_1279449
vGluT1	Synthetic peptide from rat vesicular glutamate transporter 1	Guinea pig	1:5,000	Millipore, AB5905, RRID:AB_2301751

aa, amino acids.

**TABLE 2:** Number of animals used for immunostaining (age and sex in brackets).

Figures 1–4, 8					
**CaBP**	**CD15**	*PKCα	**GlyT1**	**ChAT**	**RBPMS**
7 (4 m, 4 m, 6 f, 9 m, 11 m, 12 m, 13 m)	4 (4 m, 8 m, 9 m, 11 m)	16 (4 m, 5 f, 5 m, 7 m, 8 m, 9 m, 10 m, 11 m, 12 f, 12 m, 12 m, 12 m, 13 m, 14 f, 15 f, 15 f)	4 (9 f, 9 m, 11 m, 13 m)	4 (4 m, 9 m, 11 m, 12 m)	3 (6 f, 9 m, 11 m)

**GNB3**	**L/M-Opsin**	**S-Opsin**	**Rhodopsin**	**vGluT1**	**Parvalbumin**
5 (4 m, 6 f, 9 m, 11 m, 13 m)	4 (4 m, 9 m, 11 m, 13 m)	4 (4 m, 9 m, 11 m, 13 m)	4 (4 m, 9 m, 11 m, 13 m)	4 (4 m, 9 m, 11 m, 13 m)	3 (9 m, 11 m, 13 m)

**Figure 5 RBPMS** density		**Figure 6 RBPMS** fovea		**Figure 7 Iba1** microglia	
**Control**	**Aged**	**Control**	**Aged**	**Control**	**Aged**

7 (2 f, 2 m, 2 m, 4 m, 4 m, 5 f, 6 f)	7 (11 m, 12 m, 12 m, 12 f, 13 m, 15 f, 15 f)	4 (2 f, 2 m, 2 m, 5 f)	7 (8 m, 9 f, 10 m 12 f, 12 m, 13 m, 15 f)	2 (4 m, 6 f)	2 (12 m, 12 m)

Ages given in years; m = male, f = female. *All stainings were double labeled with CtBP2.

### Image acquisition

Following immunolabeling, retinal tissue samples were mounted in Aqua-Poly/Mount and imaged using confocal microscopy (Leica TCS SP8). Samples were scanned with HC PL APO 20×/0.70, HC PL APO 40×/1.3, or HC PL APO 63×/1.4 oil immersion objectives. Voxel size was adjusted with respect to the experimental question. Ganglion cell density was measured at defined regions of interest (ROIs) along the temporal-nasal axis. For cell counting, we used the Cell Counter plugin of ImageJ. The ROIs included a minimum of 100 RBPMS positive ganglion cells. Cone and rod densities were counted at two positions (1 mm eccentricity = central, 4 mm eccentricity = peripheral; Grünert and Martin, 2020) in 100 × 100 μm sampling fields; rod bipolar cell densities were counted at the same two positions in 200 × 200 μm sampling fields. Unless stated otherwise, projections of confocal stacks are shown. Images were adjusted in brightness and contrast and occasionally filtered for presentation purposes.

### Statistics

To quantify age-related changes, cell densities of cones, rods and rod bipolar cells were compared between control animals and old animals. For statistical analyses SigmaStat program (version 4.0, Systat Software Inc., San Jose, United States) was used. Kolmogorov-Smirnov tests were performed to check for normality and following *t*-tests or Mann-Whitney Rank Sum tests as appropriate were performed. A *p* = 0.05 was considered being significant.

## Results

### Neurons are preserved in the aged retina

**FIGURE 1:**
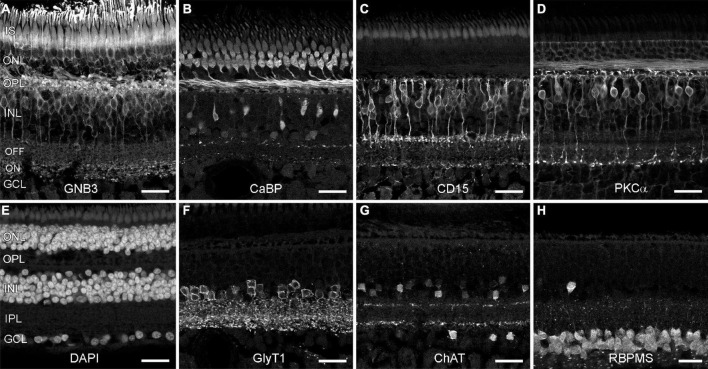
Neuronal types are preserved in old marmoset retina. Shown are vertical vibratome sections through central **(C,D,F–H)** and midperipheral **(A,B,E)** retina from aged marmosets (9 and 11 years old) that were labeled with antibodies to distinct neuron types and DAPI. **(A)** Photoreceptors and ON bipolars cells (GNB3). **(B)** Cones and one OFF bipolar cell type (calbindin). **(C)** Flat midget bipolar cells (CD15). **(D)** Rod bipolar cells (PKCα). **(E)** DAPI (all nuclear layers). **(F)** Glycinergic amacrine cells (GlyT1). **(G)** Starburst amacrine cells (ChAT). **(H)** Ganglion cells (RBPMS). IS, inner segments; ONL, outer nuclear layer; OPL, outer plexiform layer; INL, inner nuclear layer; IPL, inner plexiform layer; GCL, ganglion cell layer. Scale bars, 25 μm.

Since aging has been associated with the loss of neurons in the human retina (Gao and Hollyfield, 1992), we asked whether marmoset monkeys also lose retinal neurons with age. First, we used markers to label cell populations and subtypes in aged retinas and examined whether all cells were present in their normal positions. We used GNB3 and calbindin to label photoreceptors and bipolar cells, respectively, and DAPI to label the three nuclear layers (Figures 1A,B,E) in several retinas (see Table 2 for number and age of animals used for each marker). In macaque, GNB3 expression was only visible in cones and ON bipolar cells (Ritchey et al., 2010), whereas in human (Cuenca et al., 2018) and marmoset retina, GNB3 was expressed in both rods and cones and, most likely, in all ON bipolar cells (Figure 1A). Calbindin was strongly expressed in cones and some OFF bipolar cells and weakly in some amacrine cells (Figure 1B) as was shown previously by Chiquet et al. (2002). We also used CD15 to label OFF midget and DB6 bipolar cells and PKCα to label rod and DB4 cone bipolar cells (Weltzien et al., 2015). OFF midget (Figure 1C) and rod bipolar cells (Figure 1D) were clearly visible in the central vibratome sections shown here, whereas the axon terminals of DB4 and DB6 were easier to detect in peripheral whole mount pieces (not shown).

We next labeled glycinergic amacrine cells with GlyT1 (Figure 1F), starburst amacrine cells with ChAT (Figure 1G), dopaminergic amacrine cells with TH (not shown), and retinal ganglion cells with RBPMS (Figure 1H). In all cases, all these neuronal types were preserved; we did not find any age-related difference in the staining pattern (for comparison see Weltzien et al., 2015; Nasir-Ahmad et al., 2021).

**FIGURE 2:**
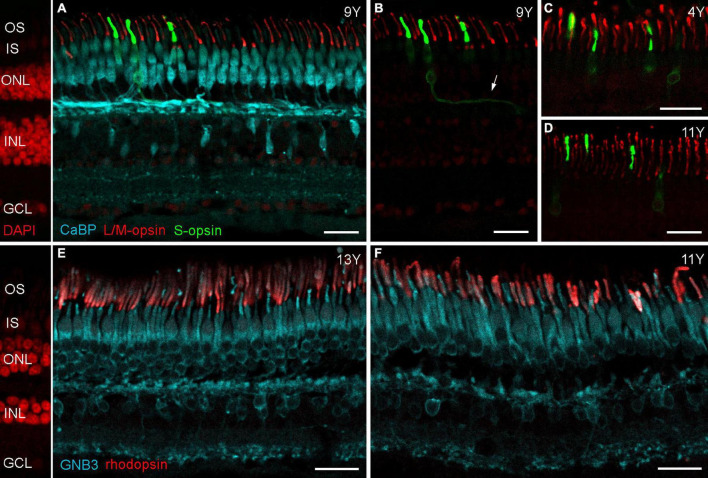
Opsin localization in aged marmoset retina. **(A)** Triple immunolabeling of calbindin, L/M-opsin and S-opsin shows that L/M-opsin expression is restricted to the outer segments of red and green cones. S-opsin is strongly expressed in outer segments, but also weakly expressed in inner segments and cell bodies of blue cones in both control **(C)** and aged marmosets **(B,D)**. The cutout in **(B)** shows the same labeled cones as in **(A)**, but without calbindin staining. The arrow points to a labeled blue cone axon. **(E,F)** Double labeling of GNB3 and rhodopsin shows no sign of opsin mislocalization: rhodopsin is only visible in the outer segments of rods. OS, outer segments; IS, inner segments; ONL, outer nuclear layer; INL, inner nuclear layer; GCL, ganglion cell layer. Scale bars, 25 μm.

To examine potential photoreceptor death more closely, we stained old and young retinas for rod and cone opsins and vGluT1. We used 1 cm long vibratome sections reaching from the fovea to the superior edge of a 4-, 9,- and a 13-year-old male, and from the fovea to the inferior edge of an 11-year-old male, and immunostained these with antibodies against L/M-opsin, S-opsin and rhodopsin. Opsin mislocalization is a clear indicator of photoreceptor degeneration (Rohrer et al., 2005; Zhang et al., 2011; Li et al., 2013) and has also been reported for the aging human retina (Yi et al., 2021) and for human retinas with retinitis pigmentosa (Fariss et al., 2000). For all marmoset sections examined here, we did not find any signs of photoreceptor degeneration: L/M-opsin was only visible in the outer segments of long and medium wavelength-sensitive (L/M or red/green) cones (Figures 2A–D) and rhodopsin in the outer segments of rod photoreceptors (Figures 2E,F). S-opsin, on the other hand, was not only strongly expressed in the outer segments of short wavelength-sensitive (blue) cones (Figures 2A–D) but also weakly in the inner segments, cell bodies, axons and cone pedicles of both young (4Y, Figure 2C) and old marmosets (9 and 11Y, Figures 2B,D). This was not surprising as other S-opsin antibodies (JH455) usually also stain the entire cone in macaque (Goodchild et al., 1996) and marmoset retina (Ghosh et al., 1997).

**FIGURE 3:**
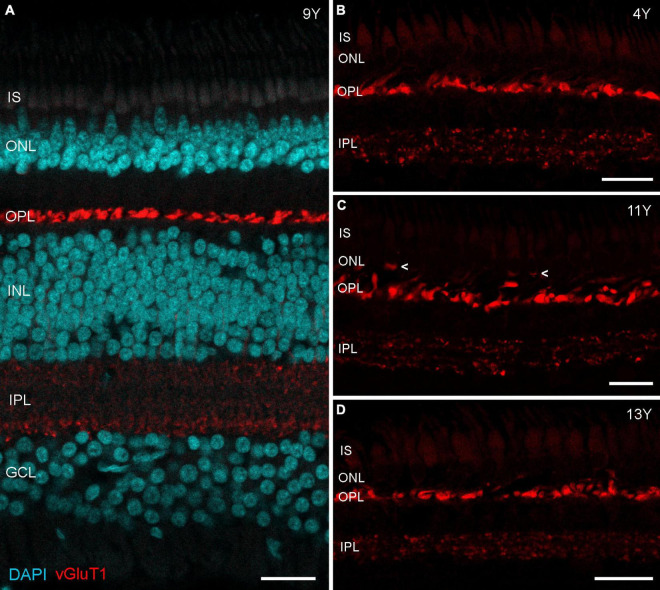
vGluT1 expression in aged marmoset retina. **(A)** vGluT1 staining in the central retina of a 9-year-old animal with strong expression in the OPL and weak expression in the IPL. **(B–D)** vGluT1 staining in the peripheral retina of a 4-year-old animal and two aged animals. Only the section of the 11-year-old animal showed some vGluT1 staining in the ONL (arrowheads in **C**), the peripheral retina of the 13-year-old animal appeared normal. IS, inner segments; ONL, outer nuclear layer; OPL, outer plexiform layer; INL, inner nuclear layer; IPL, inner plexiform layer; GCL, ganglion cell layer. Scale bars, 25 μm.

Immunostaining for vGluT1 revealed strong expression in photoreceptor terminals in the OPL and weaker expression in bipolar cell terminals in the IPL (Figure 3), as expected from previous findings in rodent and human retina (Haverkamp et al., 2003; Gong et al., 2006). Interestingly, Sullivan et al. (2007) found a redistribution of vGluT1 from the OPL into the ONL on midperipheral retina sections of human eyes affected by age-related macular degeneration (AMD). We therefore also focused on mid- to peripheral areas but found only one spot where vGluT1 appeared in the ONL of an 11-year-old male (arrowheads in Figure 3C); vibratome sections of the 13-year-old male looked the same as the control sections (Figures 3B,D). Together, opsin staining and vGluT1 expression suggest that photoreceptor degeneration is not occurring in geriatric common marmosets.

**FIGURE 4:**
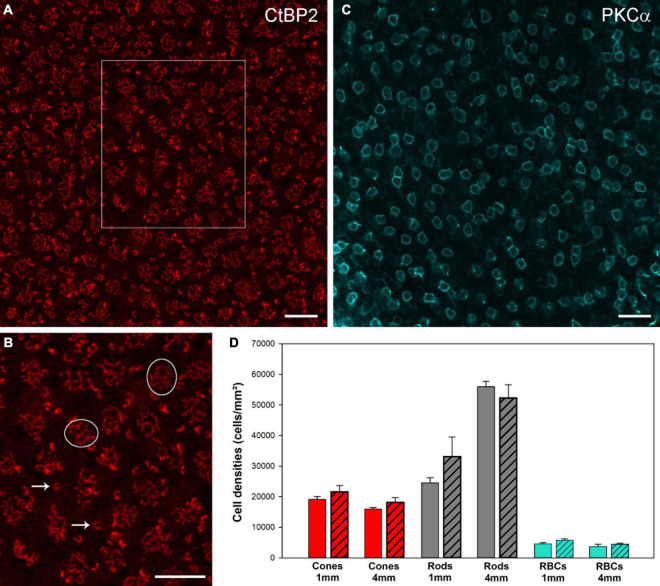
CtBP2 und PKCα immunostaining to quantify photoreceptor and rod bipolar cell densities in whole mount preparations. **(A)** CtBP2 labeling in a square of 100 × 100 μm at 4 mm temporal of a 13-year-old animal. **(B)** High-resolution image of the frame in **(A)**. **(C)** PKCα labeling in a square of 200 × 200 μm at 4 mm temporal in a 12-year-old animal. For the cones, we counted the cone pedicles that were seen as clusters of CtPB2-labeled synaptic ribbons (circles). For the rods, we counted the single horseshoe-shaped synaptic ribbons (arrows) seen outside or above the cone ribbon clusters, as it is known that each single ribbon represents one rod synaptic ending. For the rod bipolar cells, we counted the number of PKCα-labeled somata in the INL. Scale bars, **(A,B)** 10 μm and **(C)** 20 μm. **(D)** Comparison of cell densities for cones (red), rods (gray) and RBCs (cyan) from control and aged animals (patterned bars) at 1 and 4 mm temporal locations. Data are presented as mean ± SEM. No significant differences were found for pairwise comparisons between control and aged animals, respectively (pairwise *t*-tests, *p* > 0.05).

We also quantified the number of cones and rods at two positions (1 and 4 mm temporal) in retinas of six aged individuals and five adult controls. For the cones, we counted the cone pedicles that were seen as clusters of CtPB2-labeled synaptic ribbons (Figures 4A,B). For the rods, we counted the single horseshoe-shaped synaptic ribbons seen outside the cone ribbon clusters, as it is known that each single ribbon represents one rod synaptic ending (Haverkamp et al., 2001). Our cone and rod counts show remarkable inter-individual variability in the aged as well as the control animals (Supplementary Table 1). These correspond to equally large variations reported in the marmoset retina literature, both between individuals (Wilder et al., 1996) and between research groups (Troilo et al., 1993; Wilder et al., 1996; Finlay et al., 2008, Supplementary Table 2 for comparison). Irrespective of these variations, the mean cone and rod densities showed no significant differences between the aged and the control group (*p* > 0.05; see Figure 4D for mean and sd. values), supporting the conclusion that there is no photoreceptor loss in aged marmosets. The same holds true for rod bipolar cells (Figure 4C): the mean rod bipolar cell densities at 1 mm and 4 mm showed no significant changes in the aged animals compared to the control group (*p* > 0.05, Figure 4D), suggesting that there is no rod bipolar cell loss in aged marmosets.

### No significant ganglion cell loss during retinal aging

**FIGURE 5:**
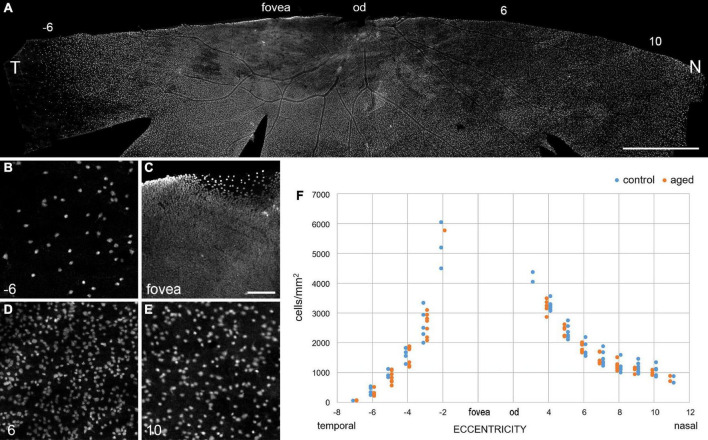
Ganglion cell density is maintained in old retinas. **(A)** Inferior retina from a 15-year-old marmoset female immunolabeled with RBPMS. **(B–E)** High-resolution images from areas along the temporal-nasal axis illustrate large changes in cell density with eccentricity (given in mm at bottom left). **(F)** Quantification of the decrease in ganglion cell density with eccentricity (distance from the fovea in mm) in aged and control retinas. Note that the ganglion cell density is much lower on the temporal side at 6 mm (–6) than on the nasal side at 6 and 10 mm. T, temporal; N, nasal; od, optic disc. Scale bars, in **(A)** 2 mm, in **(C)** 100 μm for **(B–E).**

Next, we focused on the ganglion cell layer and estimated the ganglion cell density with RBPMS immunolabeling in whole mount preparations (Rodriguez et al., 2014). In total, we labeled half retinas of seven adult animals (control; age and sex: 2 f, 2 m, 2 m, 4 m, 4 m, 5 f, 6 f) and seven old animals (aged; 11 m, 12 m, 12 m, 12 f, 13 m, 15 f, 15 f) and determined the ganglion cell density along the temporal-nasal axis. The high-resolution images in Figure 5 illustrate the large differences in cell density at various eccentricities. Because of the high density near the fovea (Figure 5C), we did not attempt to count cell numbers at eccentricities below 2 mm. Nasir-Ahmad et al. (2022) used vibratome sections of common marmosets to calculate areal densities in central retina and estimated peak values of 65,000 cell/mm^2^ at 0.57 mm and 33,000 cells/mm^2^ between 0.6 and 1.5 mm eccentricity. In our study, the ganglion cell density decreased from near 5,500 cells/mm^2^ at 2 mm to 70 cells/mm^2^ at 7 mm eccentricity in the temporal retina and from 4,300 cells/mm^2^ at 3 mm to 800 cells/mm^2^ at 11 mm eccentricity in the nasal retina (Figure 5F). The cell densities between 3 and 6 mm temporal and between 4 and 10 mm nasal showed no significant differences between control and aged animals (Table 3, *p* > 0.05 for comparisons of control vs. aged). The mean values of the aged retinas were slightly lower at some points of eccentricity and slightly higher at others.

**TABLE 3:** Ganglion cell density in control and aged animals.

Eccentricity (mm)	Control mean ± sd (cells/mm^2^)	Aged mean ± sd (cells/mm^2^)
10 (nasal)	1,070 ± 190	986 ± 79
9	1,158 ± 176	1,112 ± 84
8	1,206 ± 185	1,236 ± 154
7	1,468 ± 235	1,492 ± 196
6	1,736 ± 244	1,844 ± 140
5	2,348 ± 231	2,384 ± 173
4	3,251 ± 162	3,224 ± 236
–2 (temporal)	5,250* ± 776	5,775**
–3	2,617 ± 532	2,619 ± 383
–4	1,635 ± 223	1,593 ± 316
–5	999 ± 144	849 ± 204
–6	401 ± 133	322 ± 118

Data was obtained from N = 4–7 individuals, *N = 3, **N = 1.

### Foveal ganglion cell pattern looks similar in young and old marmosets

**FIGURE 6:**
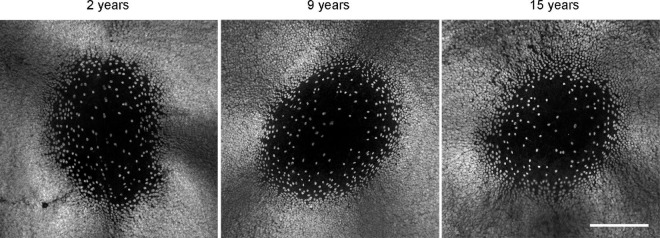
RBPMS whole mount staining reveals no qualitative difference in foveal ganglion cell pattern at different ages. Scale bar, 200 μm.

Curcio and Drucker (1993) found a 25% reduction in the number of ganglion cells around the fovea in eyes from aged humans (66–86 years of age), compared to young individuals in their mid-thirties. Although we did not quantify the ganglion cell density in the central marmoset retina, we assessed the RBPMS staining quality in and around the fovea of several animals (*n* = 4 control, *n* = 7 aged). As shown in Figure 6, tile scans and image projections of three exemplary foveae from 2, 9, and 15 years old animals did not reveal any noticeable age-related difference in the staining pattern of ganglion cells in central retina.

### No age-related changes in the distribution and density of microglia in the marmoset retina

**FIGURE 7:**
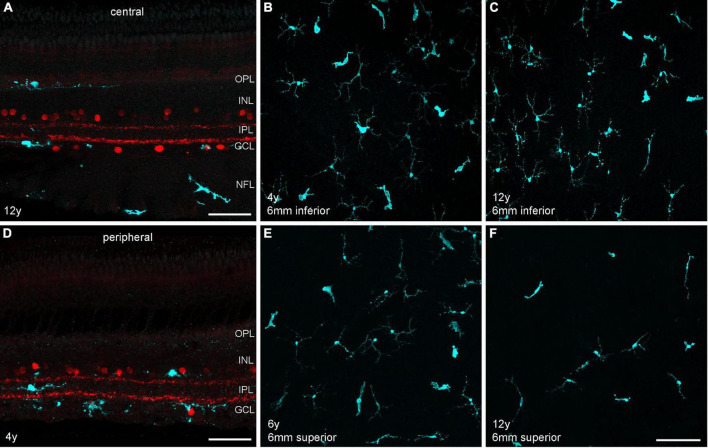
Microglia staining in different retinal areas of control and aged marmosets. **(A)** Central vibratome section of a 12-year-old male, double labeled with Iba1 (cyan) and ChAT (red). Microglia are primarily visible in the outer retina in the OPL and in the inner retina in the GCL and nerve fiber layer (NFL). **(B,C)** Whole mount projections of microglia at 6 mm inferior from control and aged marmosets (4, 12 years). **(D)** Peripheral vibratome section of a 4-year-old male, same labeling as in **(A)**. Microglia are primarily distributed in the inner retina among ChAT+ cells in the INL and GCL and in between in the IPL. **(E,F)** Whole mount projections of microglia at 6 mm superior from control and aged marmosets 6, 12 years). Scale bars, in **(A,D)** 50 μm, in **(F)** 100 μm for **(B,C)** and **(E,F)**.

Another interesting aging aspect is the potential contribution of retinal microglia to age-related changes in the macula. A recent single-cell transcriptomic study revealed an increase in microglia with retinal aging in humans (Yi et al., 2021). A study in the macaque retina showed that microglia densities in the fovea and macula, but not in the peripheral retina, were significantly higher in the aged retina in comparison to the young retina (Singaravelu et al., 2017). The authors used an antibody against the ionized calcium-binding adaptor molecule 1 (Iba1) that recognizes a protein specifically expressed by microglia among central nervous system (CNS) cells in multiple species, including marmosets (Barkholt et al., 2012). Using the same approach, we labeled central and peripheral vibratome sections and whole mount samples with Iba1 and analyzed microglia distribution and density at different areas (Figure 7) in four animals (*n* = 2 control, *n* = 2 aged). In central retina, microglia were distributed across all retinal layers from the OPL to the nerve fiber layer, whereas in peripheral retina microglia were mainly found in the inner retina in both control and aged animals (Figures 7A,B). The images in Figures 7C–F represent z-projections of confocal stacks throughout the retinal layers of flat-mounted peripheral samples (6 mm inferior and 6 mm superior). When counting the microglia in different areas we found large density differences between individuals, but no clear tendency toward higher or lower densities in the aged retinas (Supplementary Table 1). For a robust quantitative analysis, a larger sample of animals would have to be studied.

### Rod bipolar cell sprouting is minimal in old marmosets

**FIGURE 8:**
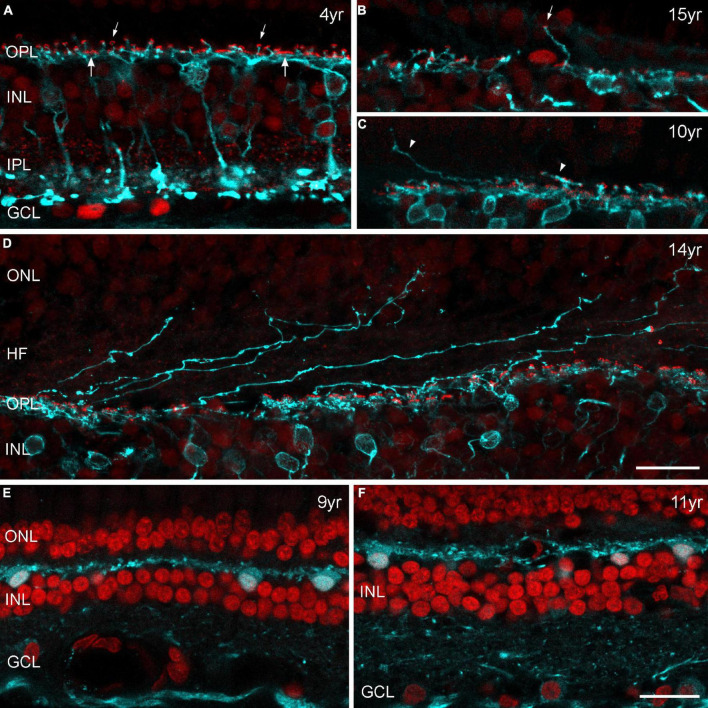
**(A–D)** Confocal images of marmoset retina from control and aged individuals double labeled for PKCα (cyan) and CtBP2 (red). PKCα immunoreactivity is visible in rod bipolar cells with axons terminating deep within the inner plexiform layer (IPL). Antibodies against CtBP2 label all photoreceptor ribbons in the outer plexiform layer (OPL) and all bipolar cell ribbons in the IPL. In the OPL, both the horseshoe-like ribbons of rod spherules (smaller arrows) and the row of individual ribbons at the cone pedicle base (larger arrows) are apparent, and all rod spherules are innervated by PKCα-labeled dendrites. Images are from vibratome sections **(A,E,F)** or from radial cut edges of whole mount preparations **(B–D)**. **(A)** Peripheral retina, control marmoset (4 years old), **(B)** example of an outgrowing dendrite with an ectopic synapse (small arrow) in the peripheral retina of a 15-year-old female. **(C)** Outgrowing dendrites (arrowheads) in the central retina of a 10-year-old male. **(D)** Central retina of a 14-year-old female with several dendritic processes projecting into the outer nuclear layer (ONL). **(E,F)** Sections of peripheral retina from two old males (9 and 11 years) labeled for parvalbumin (cyan) and DAPI (red). Parvalbumin is strongly expressed in horizontal cells in the outer retina and less strongly in ganglion cells in the inner retina. Horizontal cells display normal morphology without any sprouting events. INL, inner nuclear layer; GCL, ganglion cell layer, HF, Henle fibers. Scale bar in **(D,F)** 20 μm, for **(A–F)**, respectively.

Because of previous reports in mouse and human retina (Liets et al., 2006; Eliasieh et al., 2007; Terzibasi et al., 2009) we expected to find a large proportion of outgrowing rod bipolar cell dendrites and ectopic synapses within the ONL of each aged retina we investigated (from 6 males and 4 females). Altogether, we labeled three complete retinas (12, 14, 15 years old) and seven half retinas (10, 11, 3 × 12, 13, 15 years old) as whole mount preparations for rod bipolar cells and examined the complete tissue at high magnification. Surprisingly, four aged animals showed no signs of aberrant dendrites at all. We observed peripheral sprouts in only three individuals: We found six sprouts in the peripheral retina of a 12-year-old male and two sprouts in the peripheral retina of a 15-year-old female. One sprout was in contact with an ectopic presynaptic ribbon (Figure 8B). For comparison, see the peripheral vibratome section of a 4-year-old male (Figure 8A). We found only one additional sprout at 4 mm temporal (Supplementary Figure 1) when we quantified the number of sprouts in relation to the number of rod bipolar cell dendrites contacting rod synaptic endings in the OPL in sampling fields at 4 mm and 5 mm temporal (Supplementary Table 1). Few aberrant dendrites were found in the central retina of a 10-year-old male (Figure 8C); some more sprouts were counted at 1 mm temporal in the retina of 15-year-old-female (Supplementary Table 1). In addition, numerous very unusual outgrowing dendrites were visible in the central retina of a 14-year-old female (Figure 8D) which seemed to follow the Henle fibers over more than 100 μm. Counts in a 100 × 100 μm sampling field revealed 39 sprouts out of 297 dendrites (Supplementary Table 1).

To confirm our results of minimal sprouting events in old marmoset, we also performed horizontal cell labeling on vibratome sections. Parvalbumin is a common marker for horizontal cells in marmosets and other primates (Chiquet et al., 2005) and has been used to label horizontal cells with elongated processes that extend into the ONL in older human retinas (Eliasieh et al., 2007). We used the same sections as for opsin staining in old marmosets (Table 2) and found no evidence of outgrowing dendrites in central and peripheral retina (Figures 8E,F).

### Rod bipolar cell sprouting in aged human retina is less abundant than previously reported

**FIGURE 9:**
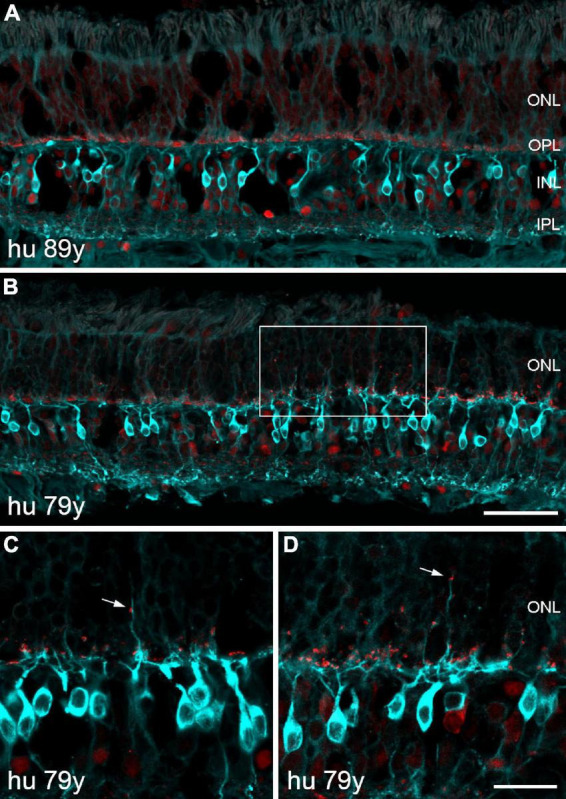
Confocal images of human retina from two old donors double labeled for PKCα (cyan) and CtBP2 (red). **(A)** Section of peripheral retina from an 89-year-old donor displaying normal morphology of rod bipolar cells and photoreceptor synapses. **(B)** Section of peripheral retina from a 79-year-old donor demonstrating outgrowing dendrites and ectopic synapses within a small area of the ONL (framed). **(C,D)** Two additional sections showing outgrowing dendrites and ectopic synapses (arrows) at higher magnification. Scale bars, **(A,B)** 50 μm and **(C,D)** 20 μm.

The absence of any severe sprouting effects in the aged marmoset retina prompted us to repeat the staining of rod bipolar and horizontal cells on sections of peripheral human retina. As expected, we did not observe any outgrowing rod bipolar cell dendrites on retinal sections of two middle-aged donors (42 and 53 years old, data not shown). For the aged human retina, we examined several peripheral retina sections (8 mm in length) from an 89-year-old donor and surprisingly found no clear examples of sprouting dendrites (Figure 9A). Retinal sections from a 79-year-old donor showed at least some small areas with outgrowing dendrites and ectopic synapses (Figures 9B–D). The same held true for horizontal cell staining with parvalbumin: no outgrowing processes in the 89-year-old retina and a few elongated processes in the 79-year-old retina (not shown). Taken together, our results in the human retina suggest that the cellular reorganization during normal aging is less severe than reported by Eliasieh et al. (2007).

## Discussion

Visual impairment is common in older people and often accompanied by significant reductions in quality of life for the affected patients. Functional studies have demonstrated several common problems such as decreased visual acuity, scotopic loss of sensitivity, and altered sensitivity to motion (Owsley, 2011). To investigate the physiological aging process, to identify possible therapeutic targets or to test new therapies, rodents are the most commonly used animal species. However, due to anatomical differences and their genetic distance to humans, translation of findings is sometimes complicated. Short-lived non-human primates like the common marmoset (*Callithrix jacchus*) start to exhibit already at the age of 6–8 years human-like aging phenotypes in a variety of organs. Thus, they might also represent a potentially interesting animal model to study age-related changes of the retina. Previous work concentrated on glaucoma-like degenerations, changes of pigments and synucleins as well as proteomic analyses. In contrast, morphological analyses are scarce and only little is known about physiological changes in the retina in healthy older animals. The aim of the current study was therefore to provide the first descriptions of age-associated changes in quantities, architecture, patterns and decline of different retinal cell populations in this animal species.

### Maintenance of photoreceptor density and cell patterns in old marmosets

Age-related decline in vision is attributed to the dysfunction and/or loss of retinal cells (Gao and Hollyfield, 1992; Curcio and Drucker, 1993). Human photoreceptor density decreases with age and rods appear to be more affected than cones. Curcio et al. (1993) showed that the density of parafoveal rods decreased significantly with increasing age, whereas the number of cones remained stable in Old world primates (Weinrich et al., 2017). Another study reported a significant loss of central cones in humans over 90 years old (Feeney-Burns et al., 1990), suggesting that cone loss may occur at very advanced ages. Panda-Jonas et al. (1995) found that photoreceptor loss was higher at an eccentricity of 5–8 mm than in the peripheral retina (14–20 mm), comparable to the results of Eliasieh et al. (2007), who estimated a higher loss of photoreceptors in the central retina (27% decrease vs. 7% in the periphery).

A recent single-cell transcriptomic study of aging human and macaque retinas revealed a dramatic decrease in rod photoreceptors and an increase in microglia with retinal aging in humans (Yi et al., 2021). Interestingly, the authors did not observe these changes in the macaque retina, which is in line with our marmoset results. Although marmosets showed age-related changes in the retinal pigment epithelium (Hadrian et al., 2019; König et al., 2019) and glaucoma-like characteristics (Noro et al., 2019), we found no evidence for age-related changes within the retina neither with regard to cell numbers nor ganglion cell pattern.

### Preserved ganglion cell number and patterns with age in marmosets

Several studies have found retinal ganglion cell loss in aging humans and rodents (humans: Gao and Hollyfield, 1992; Curcio and Drucker, 1993; Harman et al., 2000; Esquiva et al., 2017; rodents: Katz and Robison, 1986; Danias et al., 2003). Others, in agreement with us, have found that the ganglion cell population in rodents or primates does not change with age (macaque: Kim et al., 1996; rodents: Feng et al., 2007; Samuel et al., 2011; Nadal-Nicolás et al., 2018). Some of the conflicting results may reflect the problem of extrapolating absolute cell counts from sections when cell volume or cell density changes. Therefore, it was not our aim to calculate the high number of ganglion cells in central sections near the fovea, where they appear tightly packed in up to six layers; and in flatmounts, we were not able to get reliable counts below 2 mm eccentricity. Furthermore, displaced amacrine cells comprise 5–10% of the neurons in the ganglion cell layer near the fovea and 75% or more of the neurons in the periphery of the primate retina (Wässle et al., 1989; Curcio and Allen, 1990). Therefore, inclusion of displaced amacrine cells in the counts could also affect the results.

Gao and Hollyfield (1992) have studied tangential sections from human foveal and far-peripheral regions, 1–2 mm^2^ in size; Curcio and Drucker (1993) have examined flatmounts of human retinas within a region 12 mm in diameter centered on the fovea. Both studies agree that aging produces a loss of ganglion cells near the fovea, but they disagree about the extent to which there is a loss in peripheral retina. Gao and Hollyfield (1992) counted all neurons in the ganglion cell layer and did not distinguish between ganglion cells and displaced amacrine cells. They found a 40% decrease in the far-peripheral retina (13 mm temporal). Curcio and Drucker (1993) used retinal flatmounts and distinguished ganglion cells from displaced amacrine cells by morphological criteria. In their study, eyes from aged individuals had a substantially lower density of ganglion cells (25%) around the fovea and along the horizontal meridian in nasal retina (6 mm from the fovea); however, elsewhere within 6 mm eccentricity, densities were similar in young and aged eyes.

Harman et al. (2000) estimated the total number of cells in the ganglion cell layer of humans throughout life and also found a significant decrease with age. The total number of neurons was lower in older human retinas and the neuronal density was lower in most retinal regions. Interestingly, the reduction in density was less for the macular region, with a value of 0.29% per year, compared to a mean reduction of 0.53% in the whole retina. Taken together, the cell counts in the ganglion cell layer of the primate retina suggest that aging results in a relatively mild and quite variable overall loss of retinal ganglion cells in humans but not in macaques (Kim et al., 1996) and marmosets (Figure 5F and Table 3).

### No sprouting rod bipolar cell dendrites and ectopic synapses in older marmosets

Some interesting age-related alterations have recently been described in the mouse retina (Samuel et al., 2011). There was a minimal decline in the number of photoreceptors and ganglion cells, but the dendritic arbors of ganglion cells shrank with age, and size and complexity of their axonal arbors in the superior colliculus decreased with age. Other neuronal types aged in different ways: amacrine cells did not remodel detectably, whereas rod bipolar cell dendrites and horizontal cell processes sprouted into the photoreceptor layer, and here most abundantly in the peripheral retina (Liets et al., 2006; Terzibasi et al., 2009). The mechanisms driving the age-dependent growth of aberrant processes into the ONL is not completely understood but occurs most likely in response to photoreceptor death or loss of photoreceptor function (Terzibasi et al., 2009), as was shown in mutant mice lacking photoreceptor function (Dick et al., 2003; Michalakis et al., 2013).

Age-dependent growth of bipolar and horizontal cell processes into the ONL was also visible in human retina and here again, more striking in the peripheral retina (Eliasieh et al., 2007). Therefore, we were very surprised to find only minimal numbers of outgrowing dendrites in the aged marmoset retina, especially in the peripheral retina. Interestingly, Sullivan et al. (2007) found strong rod bipolar cell sprouting in human AMD retinas, but no sprouting in five normal ones, 61–85 years of age, indicating that cell sprouting is not associated with normal aging but rather symptomatic of disease. The AMD study is in line with our results on human retinal sections (Figure 9) and could explain the unusual staining pattern in the central retina of an old marmoset female in Figure 8D and the high number of sprouts at 1 mm in this and a second retina (Supplementary Table 1). We believe that we may have unknowingly stained a diseased aged retina (despite regular clinical health evaluation of the animals) rather than a normal aged retina. It is also conceivable that unknown environmental factors such as differences in ambient light exposure over the life history of the donors may affect the retina; this could explain the different amounts of aberrant sprouting seen in the aged human retinas by Eliasieh et al. (2007) and us.

## Conclusion

Altogether, these results suggest on one hand that careful considerations should be made when choosing an appropriate animal model to study specific retinal changes, and that both macaque and common marmoset as the most commonly studied non-human primate species are not always the optimal candidates for aging research depending on the research question. On the other hand, the nearly complete absence of age-related declines in various retinal cell populations together with preserved neuronal architecture and staining patterns in an animal species that highly depends on both its visual and auditory system in the wild poses the question how this species is able to maintain cellular functions in the retina up to old age. The oldest animals in the study cohort were 15 years old, which is already older than the average life span of this species and corresponds to approximately 80 years old humans. Further studies could clarify the molecular mechanisms of this phenomenon and might contribute to a deeper understanding of physiological and pathological aging processes in human and non-human primate species as well as their similarities and differences.

## Data availability statement

The original contributions presented in this study are included in the article/Supplementary material, further inquiries can be directed to the corresponding author/s.

## Ethics statement

The studies involving human participants were reviewed and approved by the Ethics Committee of the University of Tübingen. The patients/participants provided their written informed consent to participate in this study. The animal study was reviewed and approved by the institutional animal welfare committee of the German Primate Center in Göttingen and by the Lower Saxony State Office for Consumer Protection and Food Safety.

## Author contributions

SH designed the project and prepared the figures. SH, KR, LP, and MM performed the experiments. SH and LP analyzed the data. SH and MM wrote the manuscript. LP commented on the manuscript. All authors read and approved the final manuscript.
