# Supplementary material for: No evidence for age-related alterations in the marmoset retina
Source: Front Neuroanat. 2022 Sep 2;16:945295. doi: 10.3389/fnana.2022.945295 (PMC9479465; doi:10.3389/fnana.2022.945295)
Supplement: Supplementary file 1 [file Data_Sheet_1.docx]

**No evidence for age-related alterations in the marmoset retina**

**by Silke Haverkamp, Katja Reinhard, Leo Peichl, Matthias Mietsch**

**Supplemental material for quantifying the density of cone pedicles, rod terminals, and rod bipolar cells**

**
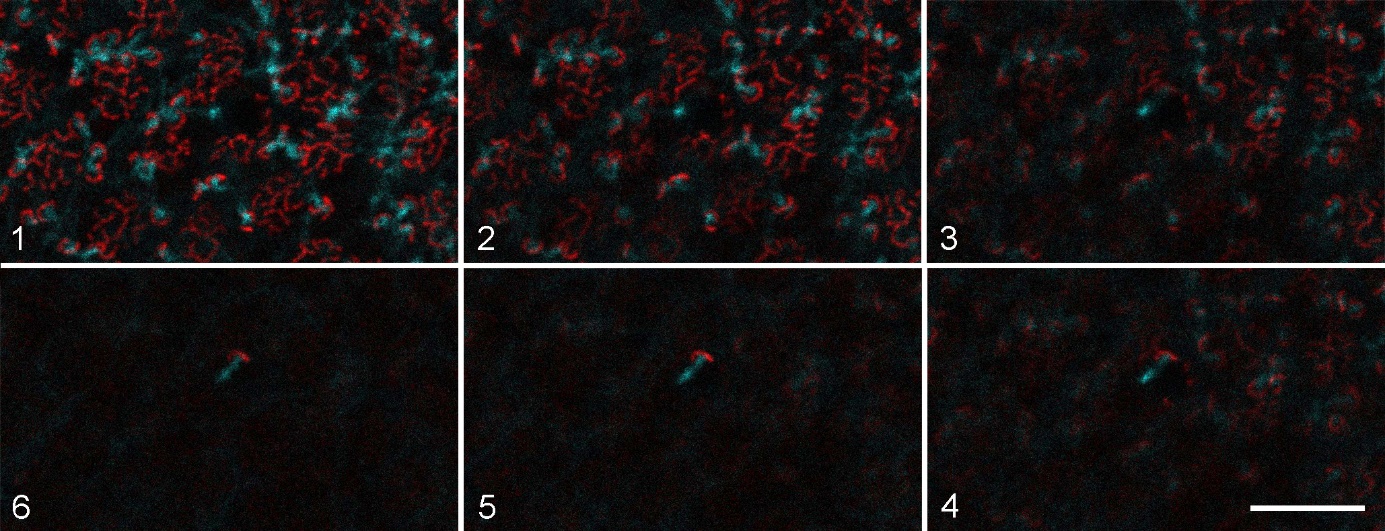
**

**Supplemental Figure 1**: CtBP2 und PKCα immunostaining to count the number of sprouts in wholemount preparations. Series of six confocal sections at high resolution showing an outgrowing rod bipolar cell dendrite (cyan) and an ectopic ribbon synapse (red) within the ONL of series 4-6. This was the only example we found at 4 mm and 5 mm temporal (see supplemental Table below). Scale bar, 10 µm.

**Supplemental Table 1. Cell densities and number of sprouts in control and aged animals**

| **Animals** | **Cones** [cells/mm^2^] | **Cones** [cells/mm^2^] | **Rods** [cells/mm^2^] | **Rods** [cells/mm^2^] | **RBCs** [cells/mm^2^] | **RBCs** [cells/mm^2^] |
| --- | --- | --- | --- | --- | --- | --- |
|  | 1 mm temporal | 4 mm temporal | 1 mm temporal | 4 mm temporal | 1 mm temporal | 4 mm temporal |
| 2m | 19600 | 16900 | 26900 | 57600 |  |  |
| 4m | 19400 | 15700 | 22000 | 51800 |  |  |
| 4m | 16300 | 14400 | 27900 | 52200 | 3775 | 2750 |
| 5m | 18300 | 17100 | 21300 | 57200 | 5150 | 5250 |
| 7m | 22000 | 15800 |  | 61000 | 4800 | 3200 |
| mean ± sd | 19120 ± 2075 | 15980 ± 1085 | 24525 ± 3357 | 55960 ± 3907 | 4575 ± 715 | 3733 ± 1333 |
|  |  |  |  |  |  |  |
| 12m | 23000 | 23500 | 45700 | 66000 | 5675 | 5700 |
| 12m | 29300 | 16200 | 58100 | 57200 | 7700 | 3750 |
| 13m | 20500 | 14500 | 23300 | 42400 | 5500 | 3925 |
| 14f | 21400 | 21200 | 29700 | 58600 | 6275 | 5225 |
| 14f | 21700 | 14600 | 25600 | 37800 | 5550 | 3775 |
| 15f | 13600 | 19000 | 16400 | 51600 | 3700 | 4600 |
| mean ± sd | 21583 ± 5030 | 18167 ± 3698 | 33133 ± 15662 | 52267 ± 10584 | 5733 ± 1332 | 4496 ± 824 |
|  |  |  |  |  |  |  |
|  | **Sprouts**  [100x100µm] | **Sprouts**  [100x100µm] | **Sprouts**  [100x100µm] |  |  |  |
|  | 1 mm temporal | 4 mm temporal | 5 mm temporal |  |  |  |
| 4m | 0/279 | 0/522 |  |  |  |  |
| 5m | 0/213 | 0/572 |  |  |  |  |
| 7m |  | 0/610 |  |  |  |  |
|  |  |  |  |  |  |  |
| 12m | 0/457 | 0/660 | 0/644 |  |  |  |
| 12m | 0/572 | 1/572 |  |  |  |  |
| 13m | 0/233 | 0/424 | 0/414 |  |  |  |
| 14f | 39/297 | 0/586 | 0/501 |  |  |  |
| 14f | 0/256 | 0/378 | 0/332 |  |  |  |
| 15f | 18/164 | 0/516 | 0/346 |  |  |  |
|  |  |  |  |  |  |  |
|  | **Microglia**  [cells/mm^2^] | **Microglia**  [cells/mm^2^] | **Microglia** [cells/mm^2^] | **Microglia** [cells/mm^2^] |  |  |
|  | 6 mm superior | 6 mm inferior | 0.5-1 mm | near ONH |  |  |
| 4m |  | 99 | 108 | 148 |  |  |
| 6f | 87 |  | 307 | 257 |  |  |
| 12m | 40 |  | 68 | 297 |  |  |
| 12m |  | 121 | 152 | 340 |  |  |

Animal ages given in years, m = male, f = female; RBC = rod bipolar cell; ONH = optic nerve head. The second line below the cell types gives the distance from the fovea (eccentricity) in mm and the retinal quadrant.

**Supplemental Table 2. Densities of marmoset cones and rods from the literature (temporal retina)**

| **Cell type** | **Density at 1 mm ecc.**  [cells/mm^2^] | **Density at 4 mm ecc.**  [cells/mm^2^] | **Source** |
| --- | --- | --- | --- |
|  |  |  |  |
| Cones mean | ~42,000 | ~32,000 | Wilder et al. 1996, Fig. 3 |
| Cones animal MY2 | ~50,000 | ~32,000 | “ |
| Cones animal MY10 | ~33,000 | ~45,000 | “ |
| Cones animal MG7 | ~24,500 | ~18,000 | Wilder et al. 1996, Fig. 15 |
| Cones | ~22,500 | ~17,000 | Finlay et al. 2008, Fig. 2A |
| Cones | ~22,500 | ~16,500 | Troilo et al. 1993, Fig. 6  (1mm=8.16°, 4mm=31.6°) |
|  |  |  |  |
| Rods | ~95,000 | ~118,000 | Wilder et al. 1996, Fig. 15 |
| Rods | ~30,000 | ~60,000 | Finlay et al. 2008, Fig. 2B |
|  |  |  |  |
| RBCs (PKCα) | 8640 | 4278 (at 3 mm) | Weltzien et al. 2015  (from vertical sections, retinal quadrant unknown) |
